# Fluid Flow and Entropy Generation Analysis of Al_2_O_3_–Water Nanofluid in Microchannel Plate Fin Heat Sinks

**DOI:** 10.3390/e21080739

**Published:** 2019-07-28

**Authors:** Hao Ma, Zhipeng Duan, Liangbin Su, Xiaoru Ning, Jiao Bai, Xianghui Lv

**Affiliations:** School of Mechanical, Electronic and Control Engineering, Beijing Jiaotong University, Beijing 100044, China

**Keywords:** pressure drop, entropy generation, nanofluids, microchannels, heat sinks, entrance effects, electronic cooling

## Abstract

The flow in channels of microdevices is usually in the developing regime. Three-dimensional laminar flow characteristics of a nanofluid in microchannel plate fin heat sinks are investigated numerically in this paper. Deionized water and Al_2_O_3_–water nanofluid are employed as the cooling fluid in our work. The effects of the Reynolds number (100 < *Re* < 1000), channel aspect ratio (0 < *ε* < 1), and nanoparticle volume fraction (0.5% < *Φ* < 5%) on pressure drop and entropy generation in microchannel plate fin heat sinks are examined in detail. Herein, the general expression of the entropy generation rate considering entrance effects is developed. The results revealed that the frictional entropy generation and pressure drop increase as nanoparticle volume fraction and Reynolds number increase, while decrease as the channel aspect ratio increases. When the nanoparticle volume fraction increases from 0 to 3% at *Re* = 500, the pressure drop of microchannel plate fin heat sinks with *ε* = 0.5 increases by 9%. It is demonstrated that the effect of the entrance region is crucial for evaluating the performance of microchannel plate fin heat sinks. The study may shed some light on the design and optimization of microchannel heat sinks.

## 1. Introduction

Recent advances in manufacturing technologies have driven the development of microelectronicmechanical systems (MEMS) [1]. The greatest challenge is overheating due to an increasing power flux and a higher thermal resistance in electronic chips [2]. Significant heat dissipation generated by the electronic chips requires a special cooling system [3]. One of the most potential applications for MEMS is the microchannel heat sink, and it has been successfully utilized for controlling the temperature in various microdevices [4].

Accurate modeling of fluid flow and heat transfer is quite important for numerous MEMS applications [5]. The recent development of microscale fluid systems has attracted much academic research of fluid flow and heat transfer in microchannels with different cross-sections [6,7,8,9,10,11,12,13,14].

Morini et al. [7] examined the effect of the viscous dissipation on the friction factor and convective heat transfer in microchannels. Liu and Garimella [8] investigated, both experimentally and numerically, liquid flow in microchannels. Onset of turbulence was verified by flow visualization in their work. Wang [10,11] investigated analytically forced convection heat transfer in rectangular ducts with various aspect ratios. Vocale et al. [12] numerically investigated the gas flow through elliptical microchannels with slip flow boundary conditions. Si Salah et al. [13] numerically studied the flow in two-dimensional rectangular microchannels using the control volume finite element method without pressure correction.

Several investigations on the developing flow in microchannels have been carried out [15,16,17,18,19,20,21,22]. Wen and Ding [16] pointed out the remarkable enhancement of convective heat transfer utilizing nanofluids, particularly in the entrance region. Mishan et al. [17] highlighted the importance of entrance effects in evaluating of the performance micro-scale heat sink. Renksizbulut et al. [20] studied numerically gas flow and heat transfer in the entry region of rectangular microchannels with velocity slip conditions.

In recent years, a great number of studies have been conducted utilizing innovative geometries [23,24,25,26,27,28,29,30,31,32,33], employing liquid coolants with excellent thermal features [34,35,36,37,38,39,40,41], and applying micro-pin-fins [42,43,44] in order to improve the capabilities of microchannel heat sinks (MCHS) removing the heat generated by electronic chips.

Pang et al. [23] proposed an optimized cooling structure for avionics applications using a multiobjective optimal design method. Vinodhan and Rajan [24] numerically investigated the overall performance of four new microchannel heat sink configurations. Lu and Vafai [25] carried out a comparative study of MCHS with different layers. Wang et al. [26] performed a numerical research on the thermal performance of MCHS with a series of trapezoidal grooves. Soleimanikutanaei et al. [31] carried out a numerical study of the heat transfer enhancement through utilizing transverse microchannels in heat sinks. Al Siyabi et al. [32] conducted an experimental research on the performance of a multilayered microchannel heat sink. Deng et al. [33] numerically examined the behavior of double-layered microchannel heat sinks with different cross-sectional shapes. 

Alternative coolants have been required due to high rate of the heat generated by increasingly powerful electronics. Xie et al. [34] presented a numerical analysis on the heat transfer and friction characteristics of a minichannel heat sink using water. Nasiri et al. [37] numerically investigated the entropy generation in MCHS with Fe_3_O_4_–water. Bahiraei and Heshmatian [38] pointed out that nanofluids containing grapheme and silver nanoparticles result in excellent thermal characteristics. Sarafraz et al. [41] evaluated the thermal performance of MCHS with rectangular microchannel employing silver–water nanofluid. Milanese et al. [45] and Iacobazzi et al. [46] presented important mechanisms of high thermal conductivity of nanofluids. Iacobazzi et al. [47] investigated the effect of clustering phenomenon on thermal conductivity of Al_2_O_3_-water nanofluid. Duan et al. [48] focused on sphere drag and heat transfer, and their work is of great significance for modeling nanofluid flow. Fan et al. [42] proposed a novel cylindrical oblique fin minichannel heat sink fitted over cylindrical heat sources. Kanargi et al. [43] investigated the behavior of an air-cooled, planar, oblique-finned heat sink for two oblique angles.

Ribs mounted in MCHS generally result in a heat transfer enhancement [49]. Khan et al. [50] conducted a three-dimensional numerical simulation of MCHS with ribbed channels in various configurations. Chai et al. [51] carried out a numerical analysis of the thermal performance of an interrupted microchannel heat sink with ribs in the transverse microchambers. 

Efficiency enhancement of thermal systems is a concern for engineers. Entropy generation minimization [52,53] and the principle of least action [54,55,56] are two important methods to study the performance optimization of a thermal system. Some efforts have been made to research into the entropy generation of different thermal systems [23,37,57,58,59,60,61,62,63,64,65,66,67,68]. Khan et al. [59] studied the performance of MCHS using an entropy generation minimization procedure, and proposed a general expression evaluating irreversibilities. Awad [60] amply reviewed thermodynamic optimization studies of microchannels based on entropy generation analysis. Some suggestions for future work were put forward by Lorenzini and Mahian [67] in the field of entropy generation in nanofluid flows.

Flow in the channels of MCHS usually cannot reach the fully developed regime. However, researchers and engineers have generally assumed microchannel heat sink flows to be fully developed, ignoring entrance effects. A literature survey demonstrates a detailed analysis of MCHS nanofluids entrance flows has not yet been reported. This paper concentrates on the pressure drop and entropy generation characteristics of microchannel plate fin heat sinks operated with nanofluids considering entrance effects.

The purpose of this study is to investigate the flow and entropy generation characteristics on three-dimensional developing laminar flow of Al_2_O_3_-water nanofluid in microchannel heat sinks with various aspect ratios of rectangular channels.

In this research, three-dimensional numerical models have been established to evaluate the laminar flow and entropy generation characteristics in microchannel plate fin heat sinks. The effects of the Reynolds number, channel aspect ratio, and volume fraction of Al_2_O_3_–water nanofluid on pressure drop and entropy generation characteristics in microchannel plate fin heat sinks are analyzed.

## 2. Mathematical Model

### 2.1. Physical Model and Assumptions

**Figure 1:**
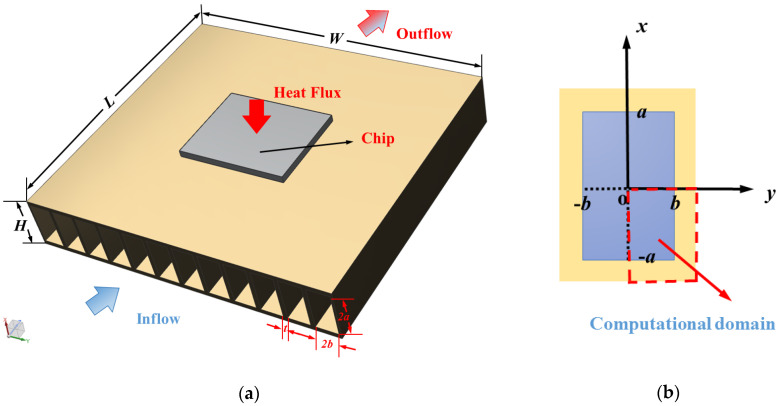
Schematics of the microchannel plate fin heat sink. (**a**) Microchannel plate fin heat sink and (**b**) rectangular channel.

The physical model of a microchannel plate fin heat sink is shown in Figure 1a. The length of the heat sink is *L*, the width is *W*, and the height is *H*. The top surface is insulated and the bottom surface is uniformly heated. As the coolant passes through the rectangular microchannels along the *z* axis, it removes the heat generated by the electronic component attached below. Due to good thermophysical properties and high economy of Al_2_O_3_–water nanofluid, most researchers utilized them as research objects [69]. Hence, deionized water and Al_2_O_3_–water nanofluid were employed as the cooling fluid in our work. There are *N* rectangular channels, and each channel has a height 2*a* and width 2*b*. The thickness of each fin is *t*. 

Taking advantage of the symmetry, a quarter of the rectangular channel as shown in Figure 1b is chosen as the numerical model to reduce computation cost. The nanofluid can be treated as incompressible Newtonian fluid and the thermophysical properties are assumed to be constant in this study [70,71]. The dimensions of the computational domain in this work are presented in Table 1.

The hydraulic diameter of channels *D_h_* is held constant and the length *L* is 14 mm. The width and height of the channel are varied with aspect ratios. The aspect ratio *ε* of a rectangular channel is defined as

(1) $$\varepsilon =\frac{b}{a}$$

and the hydraulic diameter of a rectangular channel is defined as

(2) $$D_{h}=\frac{4A}{P}=\frac{2ab}{a+b}$$

### 2.2. Governing Equations

The continuum approach is valid in this study [1]; the continuity and momentum equations are solved numerically considering nanoparticle volume fraction. The governing transport equations of mass and momentum are as follows.

Continuity equation:

(3) $$\nabla \cdot \vec{U}=0$$

Momentum equation:

(4) $$\rho_{nf}\vec{U}(\nabla \cdot U_{i})=-\nabla P+\mu_{nf}\nabla^{2}U_{i},\;i=1,2,3$$

A uniform velocity profile is given at the inlet and no counterflow at the outlet.

### 2.3. Thermophysical Properties

Single phase flow of Al_2_O_3_–water nanofluid with four different volume fractions (ϕ = 0.5, 1, 3, and 5 percent) is employed in current work. Based on the properties of deionized water and Al_2_O_3_ particles tabulated in Table 2, the effective density, specific heat capacity [72], viscosity [73], and thermal conductivity [74] are calculated using the following expressions.

(5) $$\rho_{nf}=\left(1-\phi \right)\rho_{bf}+\phi \rho_{p}$$

(6) $${\left(\rho C_{p}\right)}_{nf}=\left(1-\phi \right){\left(\rho C_{p}\right)}_{bf}+\phi {\left(\rho C_{p}\right)}_{p}$$

(7) $$\frac{\mu_{nf}}{\mu_{bf}}={\left(1-\phi \right)}^{-2.5}$$

(8) $$\frac{k_{nf}}{k_{bf}}=1+64.7\phi^{0.7460}{\left(\frac{d_{bf}}{d_{p}}\right)}^{0.3690}{\left(\frac{k_{p}}{k_{bf}}\right)}^{0.7460}Pr^{0.9955}Re^{1.2321}$$

where *k*, *μ*, *ρ*, and *Cp* denote the thermal conductivity, dynamic viscosity, density, and specific heat capacity, respectively. The subscripts *bf*, *p*, and *nf* represent base fluid (deionized water), nanoparticle, and nanofluid, respectively. The simulation parameters of thermophysical properties of Al_2_O_3_–water nanofluid are presented here in Table 3.

### 2.4. Fluid Flow Analysis

Applying the method of scale analysis to compare the force scale between friction and inertial forces, the nondimensional channel length *ξ* can be obtained:

(9) $$\frac{\mu \frac{\partial^{2}u}{\partial y^{2}}}{\rho u\frac{\partial u}{\partial x}}\sim \frac{\mu \frac{U}{D_{h}^{2}}}{\frac{\rho U^{2}}{L}}=\frac{L}{D_{h}Re_{D_{h}}}=\xi$$

When *ξ* >> 1, the fully developed flow momentum equation in rectangular channels is expressed as

(10) $$\frac{\partial^{2}u}{\partial x^{2}}+\frac{\partial^{2}u}{\partial y^{2}}=\frac{1}{\mu }\frac{dp}{dz}$$

The Fanning friction factor *f* is given by the following equation.

(11) $$f=\frac{\Delta P}{2\rho u_{m}^{2}z/D_{h}}$$

For fully developed laminar flow in rectangular channels, Shah and London [75] provided the expression of the friction factor Reynolds number product as follows

(12) $$fRe=24\left(1-1.3553\varepsilon +1.9467\varepsilon^{2}-1.7012\varepsilon^{3}+0.9564\varepsilon^{4}-0.2537\varepsilon^{5}\right)$$

Further, a more accurate theoretical solution was developed by Duan and Muzychka [76,77], and is expressed as

(13) $$fRe_{D_{h}}=\frac{24}{{\left(1+\varepsilon \right)}^{2}\left[1-\frac{192\varepsilon }{\pi^{5}}\left(\tanh\left(\frac{\pi }{2\varepsilon }\right)+\frac{1}{243}\tanh\left(\frac{3\pi }{2\varepsilon }\right)\right)\right]}$$

Flow in the channels of MCHS usually cannot reach the fully developed regime. Considering the developing region, the pressure drop equations are expressed in terms of an apparent friction factor as follows

(14) ΔP=2(fappRe)μumzDh2

### 2.5. Entropy Generation Analysis

The analysis of entropy generation to evaluate the behavior of thermal devices is a practical technique. The volumetric entropy generation rate ${\dot{S}}_{\mathrm{gen},t}^{\prime \prime \prime }$ can be presented as [78]

(15) $${\dot{S}}_{\mathrm{gen},t}^{\prime \prime \prime }={\dot{S}}_{\mathrm{gen},h}^{\prime \prime \prime }+{\dot{S}}_{\mathrm{gen},f}^{\prime \prime \prime }$$

where ${\dot{S}}_{\mathrm{gen},h}^{\prime \prime \prime }$ and ${\dot{S}}_{\mathrm{gen},f}^{\prime \prime \prime }$ are the 3D volumetric thermal and viscous entropy generation rates, respectively, and are expressed as [78]

(16) $${\dot{S}}_{\mathrm{gen},h}^{\prime \prime \prime }=\frac{k_{nf}}{T^{2}}\left({\left(\frac{\partial T}{\partial x}\right)}^{2}+{\left(\frac{\partial T}{\partial y}\right)}^{2}+{\left(\frac{\partial T}{\partial z}\right)}^{2}\right)$$

(17) $${\dot{S}}_{\mathrm{gen},f}^{\prime \prime \prime }=\frac{\mu_{nf}}{T}\left[2\left({\left(\frac{\partial u}{\partial x}\right)}^{2}+{\left(\frac{\partial v}{\partial y}\right)}^{2}+{\left(\frac{\partial w}{\partial z}\right)}^{2}\right)+{\left(\frac{\partial u}{\partial y}+\frac{\partial v}{\partial x}\right)}^{2}+{\left(\frac{\partial u}{\partial z}+\frac{\partial w}{\partial x}\right)}^{2}+{\left(\frac{\partial w}{\partial y}+\frac{\partial v}{\partial z}\right)}^{2}\right]$$

The global entropy generation rates are given by the integration of the volumetric entropy generation rates over the whole domain as follows

(18) $${\dot{S}}_{\mathrm{gen},h}=\int {\dot{S}}_{\mathrm{gen},h}^{\prime \prime \prime }dV$$

(19) $${\dot{S}}_{\mathrm{gen},f}=\int {\dot{S}}_{\mathrm{gen},f}^{\prime \prime \prime }dV$$

(20) $${\dot{S}}_{\mathrm{gen},t}=\int {\dot{S}}_{\mathrm{gen},t}^{\prime \prime \prime }dV$$

The total entropy generation rate can be conveniently calculated by the following expression [59].

(21) $${\dot{S}}_{\mathrm{gen},t}=\dot{Q}\left[\frac{1}{T_{a}}-\frac{1}{T_{b}}\right]+\frac{\dot{m}\Delta P}{\rho T_{a}}$$

where *T_a_*, *T_b_*, and $\dot{Q}$ represent the ambient temperature, the temperature of the heat sink base and heat transfer rate, respectively. Substituting Equation (14) into Equation (21), the global entropy generation rate considering entrance effects is obtained as follows

(22) S˙gen,t=Q˙[1Ta−1Ta]+2(fappRe)m˙μumzρTaDh2

## 3. Numerical Method

A computational fluid dynamics software, ANSYS Fluent 18.0, which can be applied to facilitate the investigation of fluid flow characteristics in microchannels [8], was employed to solve the governing equations. Uniform inlet velocity is specified and uniform inlet temperature of the working fluid is set to 290 K. Moreover, zero relative pressure is utilized on the outlet and no slip velocity boundary conditions are applied to the walls. The SIMPLE algorithm was adopted to deal with the coupling between velocity and pressure. Considering the accuracy of calculations, double precision was used and second-order upwind scheme was applied on momentum equations. The convergence criterion for the residuals of the continuity equation and momentum equations were less than 1 × 10^−9^.

The whole computational domain was meshed using hexahedral elements and performed in structured grids. The mesh in the streamwise direction had a double successive ratio of 1.013 near the entrance and 1.005 near the outlet. The grid sensitivity test was conducted for more accurate numerical results with less computational cost. The comparison of three different grid density distributions of 500 × 20 × 20, 700 × 30 × 30, and 900 × 40 × 40 were established at the aspect ratio *ε* = 1 of rectangular fluid domain. The *f*_app_*Re*, at the entrance region, changed by 1.52% from the first to the second mesh, and only varied by less than 0.65% upon further refinement to the finest grid. In view of the calculation accuracy and time cost, the second one was chosen as the grid to get mesh independent solution. The meshes of other aspect ratios had also been tested and the mesh quantities are shown in Table 1.

## 4. Results and Discussion

### 4.1. Model Validation

**Figure 2:**
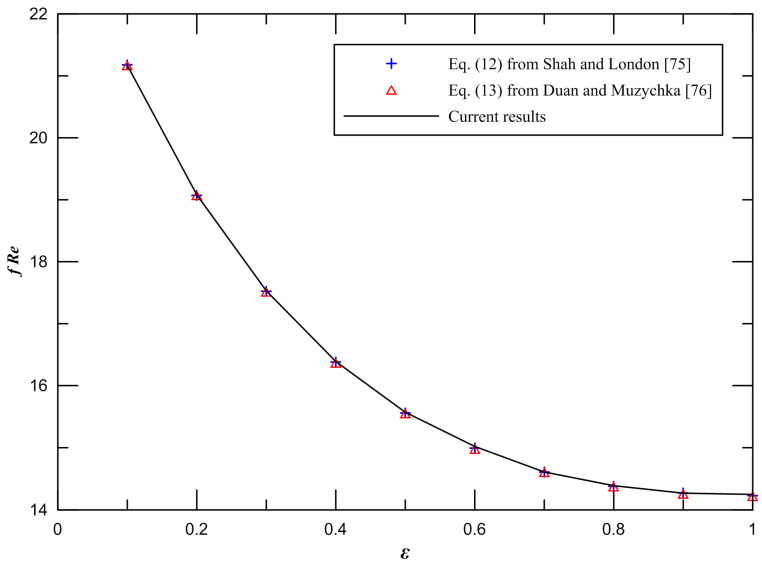
Comparison of *fRe* for Shah and London [75] and Duan and Muzychka [76] at an aspect ratio of 1.

Firstly, in order to verify the correctness of the numerical method, the numerical simulations of ten different aspect ratios of rectangular microchannels are implemented. The results of Poiseuille number in the fully developed laminar flow of rectangular channels is obtained by numerical calculation. Figure 2 demonstrates the comparison between the current results and the available numerical data from Shah and London [75] and the available analytical data from Duan and Muzychka [76]. It is found that the difference between the current results and available data from Shah and London [75] is less than 0.2%. The maximum difference between the current results and available data from Duan and Muzychka [76] is less than 0.3%.

**Figure 3:**
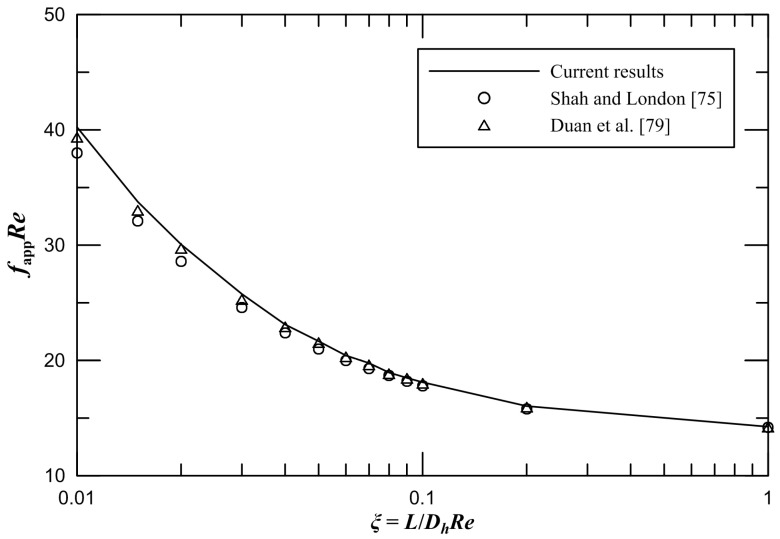
Comparison of *f*_app_*Re* for Shah and London [75] and Duan et al. [79] at an aspect ratio of 1.

Secondly, we pay attention to friction characteristics of the entry region of rectangular microchannels. It is presented that the comparison between the obtained results of the apparent friction factor and the available results from Shah and London [75] and Duan et al. [79] when the aspect ratio *ε* = 1 in Figure 3. It is observed that our numerical results agree with the available numerical data [75,79] quite well. The maximum difference between the current results and the available numerical data is less than 5.9%, which does prove the numerical means used is valid.

### 4.2. Effect of Reynolds Number on Pressure Drop and Frictional Entropy Generation

**Figure 4:**
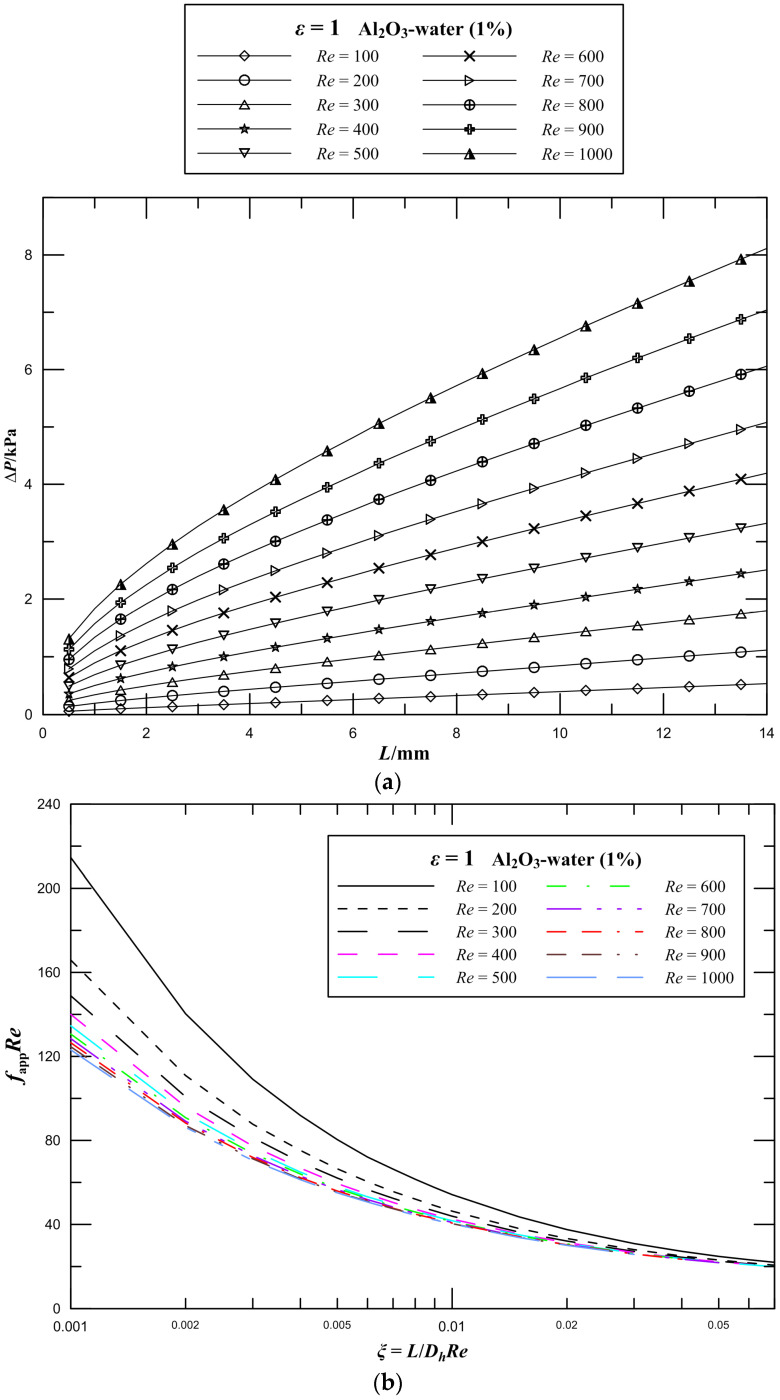
Effect of Reynolds number on flow parameters for rectangular channels. (**a**) Pressure drop; (**b**) *f*_app_*Re*.

For the 1 vol.% Al_2_O_3_–water nanofluid, three-dimensional numerical simulations of rectangular microchannel heat sinks with aspect ratio *ε* = 1 were carried out in the range of 100 < *Re* < 1000. The pressure drop parameter as a function of Reynolds number is shown clearly in Figure 4a. It is seen that the pressure drop monotonically increases along the flow direction in the microchannel heat sink, and the pressure drop observably increases as Reynolds number increases for the same aspect ratio. When the Reynolds number increases from 500 to 1000, the pressure drop of the channel increases by 144%. An increase in the flow velocity of the working fluid leads to a remarkable increase in the flow resistance, thus resulting in a significant pressure drop. Furthermore, it is obviously observed that the higher pressure gradient occurs where *L* is just less than 4 mm, which corresponding to nondimensional flow distance ranges from 0.01 to 0.1 for different Reynolds numbers, and the pressure drop increases proportionately later along the flow direction in channels for the same Reynolds number. 

The effect of Reynolds number on *f*_app_*Re* values of laminar flow in microchannel plate fin heat sinks has been studied first in this work. The variations of *f*_app_*Re* in different Reynolds numbers with the nondimensional flow distance are illustrated in Figure 4b. From the figure, it is seen that the *f*_app_*Re* has a rapid reduction with an increase in *ξ* and the *f*_app_*Re* at the entrance region is significantly higher than the fully developed values of *fRe*. The *f*_app_*Re* is found to be sensitive to low Reynolds numbers. Moreover, the *f*_app_*Re* at low Reynolds numbers is markedly higher than the values at high Reynolds numbers, especially in the entrance region. 

**Figure 5:**
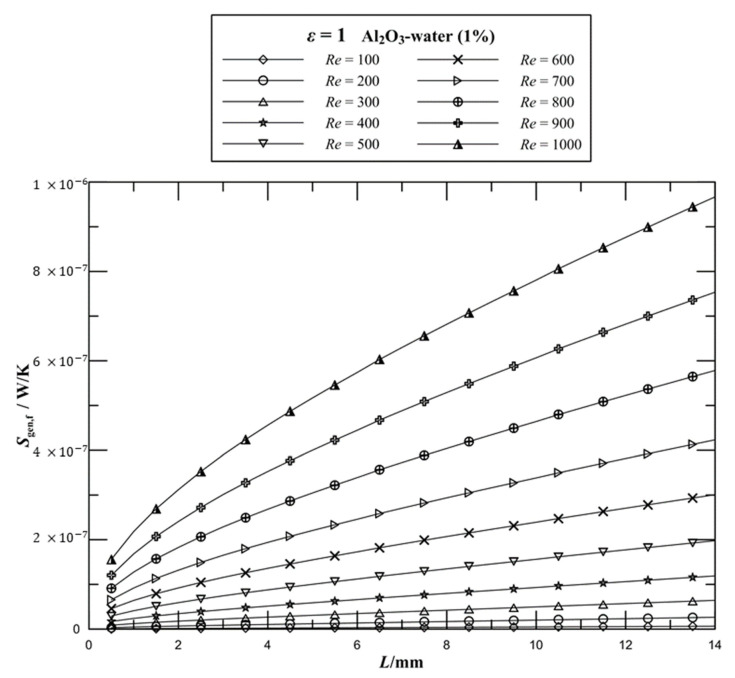
Effect of Reynolds number on the frictional entropy generation rate for rectangular channels.

The variations of the frictional entropy generation rate with different *Re* numbers are shown in Figure 5. It can be seen that for a given volume fraction of Al_2_O_3_–water nanofluid, the frictional entropy generation rate dramatically increases by increasing the Reynolds number. When the Reynolds number increases from 100 to 200, the frictional entropy generation rate increases approximately by three times. Moreover, when the Reynolds number increases from 500 to 1000, the frictional entropy generation rate of the channel increases by 389%. This increment is significant at higher values of Reynolds number. An increase in the flow velocity of the working fluid leads to the increase in the flow resistance so that more pressure energy needs to be supplied to drive the flow in MCHS, which increases the frictional dissipation and irreversibility, leading to a significant increase in entropy generation. In addition, the addition of nanoparticles in the working fluid further increases the entropy generation. It is observed that the higher entropy generation gradient in the entry region, which displays the entrance effects. 

### 4.3. Effect of Aspect Ratio on Pressure Drop and Frictional Entropy Generation

**Figure 6:**
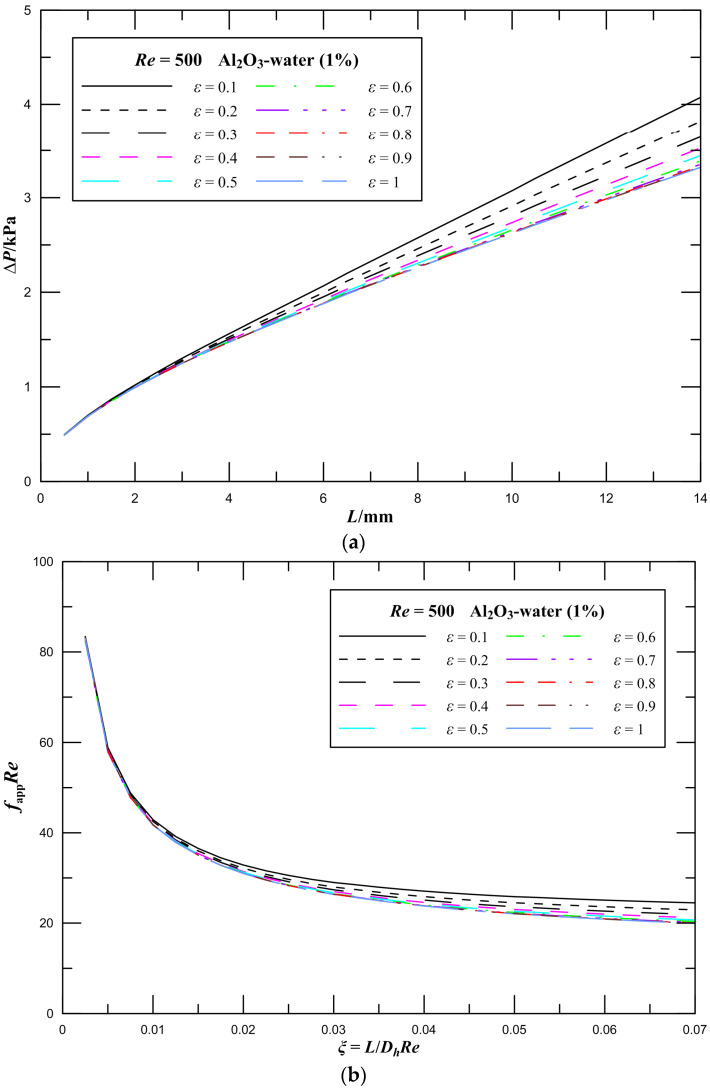
Effect of aspect ratio on flow parameters for rectangular channels. (**a**) Pressure drop; (**b**) *f*_app_*Re*.

For the 1 vol.% Al_2_O_3_–water nanofluid, three-dimensional numerical simulations of rectangular microchannel heat sinks with various aspect ratios were conducted at a Reynolds number of 500. Figure 6a illustrates the pressure drop of microchannel heat sinks with aspect ratios ranging from 0.1 to 1. As can be observed, the pressure drop monotonically increases along the flow direction in the microchannel heat sink, and the pressure drop slightly increases as the aspect ratio decreases for the same Reynolds number. The pressure drop of the channel is higher at lower values of *ε*. In detail, when the aspect ratio increases from 0.6 to 1, the pressure drop of the channel decreases by 2%; however, when the aspect ratio increases from 0.1 to 0.5, the pressure drop of the channel decreases by 15%. This is because as the aspect ratio increases, the circumferential area of rectangular microchannels decreases, that is, the contact area of the working fluid with the walls of the channels decreases. Thus, the flow resistance decreases, which results in a slight decrease in the pressure drop. Especially at low aspect ratios, the reduction in the contact area is greater. Likewise, it is observed that the higher pressure gradient in the entrance region of rectangular channels. In the entrance region, the high velocity gradient at the wall results in a high shear stress and the high pressure gradient needed to produce the high acceleration in the near wall region.

**Figure 7:**
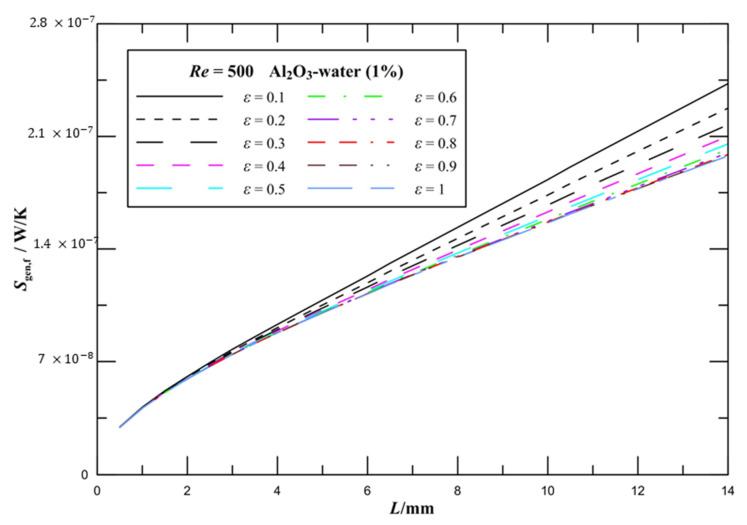
Effect of aspect ratio on the frictional entropy generation rate for rectangular channels.

The effect of aspect ratio on *f*_app_*Re* values of laminar flow in microchannel plate fin heat sinks is illustrated in Figure 6b. There is a great reduction in *f*_app_*Re* where *ξ* is less than 0.04, and subsequently *f*_app_*Re* tends to a certain value. It is observed that the effect of aspect ratio on *f*_app_*Re* values is evident when *ξ* is greater than 0.04, and the influence increases by increasing *ξ*. The effect of aspect ratio on the frictional entropy generation rate is presented in Figure 7. It is noted that the frictional entropy generation rate is higher for lower aspect ratios. The frictional entropy generation rate decreases with increasing *ε*. The influence of aspect ratio on the frictional entropy generation rate is unobvious at larger aspect ratios. Figure 6 and Figure 7 illustrate that the pressure drop and frictional entropy generation are not observably affected by the aspect ratio at the entrance region. The reason is the variation in the contact area caused by the change in the aspect ratio has not yet worked at the entry region, and as the length of the channels increases, the effect of the aspect ratio is accumulated and displayed. 

### 4.4. Effect of Volume Fraction of Al_2_O_3_-water Nanofluid on Pressure Drop and Frictional Entropy Generation 

**Figure 8:**
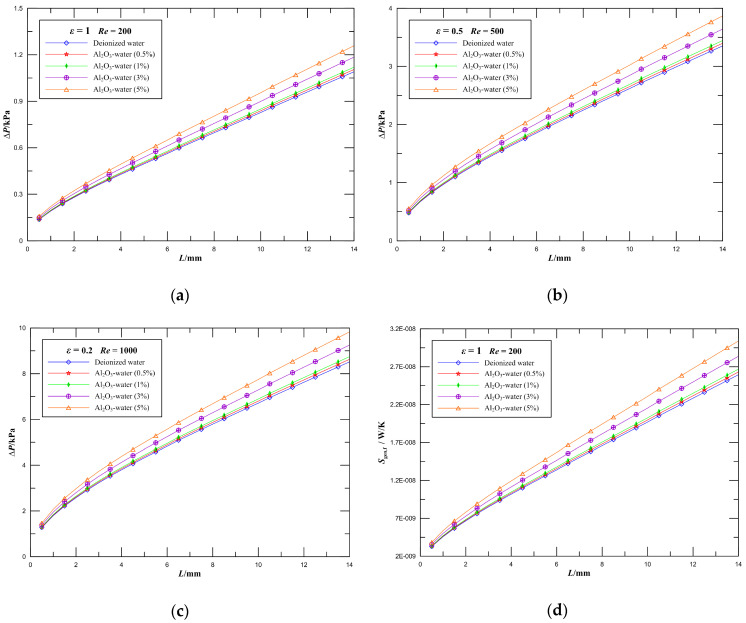
Effect of nanoparticle volume fraction on pressure drop and frictional entropy generation rate. (**a**) Pressure drop of rectangular microchannel heat sinks with *ε* = 1 at *Re* = 200; (**b**) Pressure drop of rectangular microchannel heat sinks with *ε* = 0.5 at *Re* = 500; (**c**) Pressure drop of rectangular microchannel heat sinks with *ε* = 0.2 at *Re* = 1000; (**d**) Frictional entropy generation rate of rectangular microchannel heat sinks with *ε* = 1 at *Re* = 200.

Numerical simulations of fluid flow in microchannel plate fin heat sinks using 0, 0.5, 1, 3, and 5 vol.% Al_2_O_3_–water nanofluid at *Re* = 200 and *ε* = 1, *Re* = 500 and *ε* = 0.5, and *Re* = 1000 and *ε* = 0.2 were carried out. Figure 8a–c illustrates the variations of the pressure drop parameter along the streamwise direction in rectangular ducts at different nanoparticle volume fractions. It is seen that the pressure drop increases along the flow direction in the microchannel heat sink, and the pressure drop slightly increases as nanoparticle volume fraction increases for the same aspect ratio. In detail, when the nanoparticle volume fraction increases from 0 to 1% at *Re* = 500, the pressure drop of rectangular microchannel heat sinks with the aspect ratio *ε* = 0.5 increases by 3%. Further, when the nanoparticle volume fraction increases from 1 to 5%, the pressure drop of the channel increases by 12%. The effect of increasing the nanoparticle concentration is to increase the pressure drop of the channel. In addition, it is obvious that the higher pressure gradient occurs when *L* is just less than 4 mm, and the pressure drop increases linearly later along the flow direction in channels for the same Reynolds number, especially in the lower aspect ratio and higher Reynolds number. The higher pressure gradient is caused by the momentum flux variation due to the change of velocity field from a uniform profile at the inlet to a specific profile downstream in the channel.

The frictional entropy generation characteristics of rectangular microchannel heat sinks with the aspect ratio *ε* = 1 at a Reynolds number of 200 were presented in Figure 8d. As can be observed, the viscous entropy generation increases mildly by increasing the nanoparticle volume fraction. When the nanoparticle volume fraction increases from 0 to 1%, the viscous entropy generation of the channel increases by 3%. Further, when the nanoparticle volume fraction increases from 0 to 5%, the viscous entropy generation of the channel increases by 17%. High nanoparticle volume fraction causes better heat transfer performance as well as higher entropy generation. The reason is that the viscosity increases with an increase in the concentration, which intensifies the frictional entropy generation and pressure drop. The contribution of the developing region plays a key role in determining the variations of the frictional entropy generation and pressure drop.

**Figure 9:**
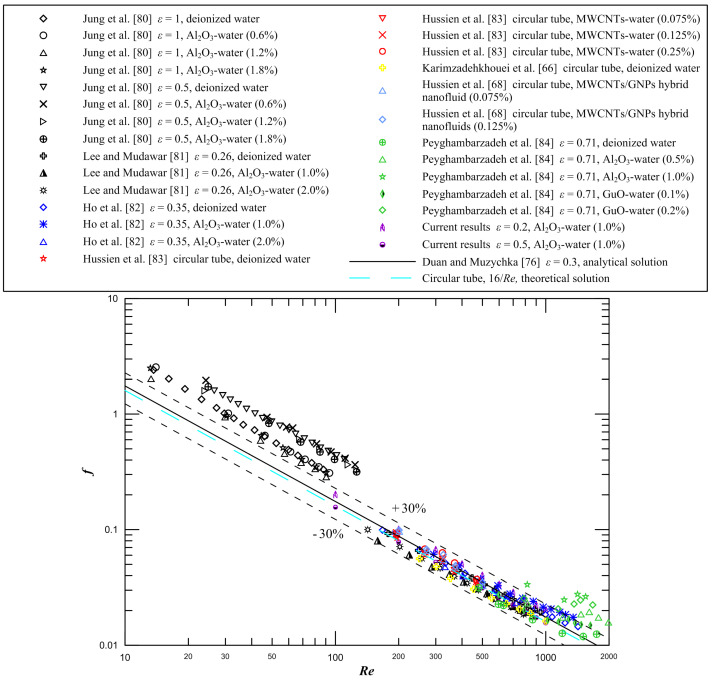
Comparison of the friction factor for current results and experimental results from the literature.

Figure 9 demonstrates the comparison of the Fanning friction factor between the current results and the available experimental results from Karimzadehkhouei et al. [66], Hussien et al. [68], Jung et al. [80], Lee and Mudawar [81], Ho et al. [82], Hussien et al. [83], and Peyghambarzadeh et al. [84]. It is observed that our results agree favorably with the experimental data reported by Karimzadehkhouei et al. [66], Lee and Mudawar [81], Ho et al. [82], and Hussien et al. [83], and our results are close to the available analytical data from Duan and Muzychka [76]. Most of the experimental data from Peyghambarzadeh et al. [84] are close to the theoretical solution. The measured values of the friction factor from Jung et al. [80] are larger than those predicted by the theoretical solution. The deviation is most likely due to entrance effects, and possibly due to measuring experimental error and channel surface roughness effects caused by the fabrication technique. Therefore, it is necessary to pay attention to entrance effects, especially in short microchannel heat sinks. Lee and Mudawar [81] and Ho et al. [82] experimentally studied the convective heat transfer and fluid flow of Al_2_O_3_–water nanofluid in rectangular microchannels with the aspect ratio *ε* = 0.26 and *ε* = 0.35, respectively, and it is seen that their results agree well with the analytical solution of rectangular microchannels with the aspect ratio *ε* = 0.3 from Duan and Muzychka [76]. The experiments of fluid flow in a circular mini-tube were carried out by Hussien et al. [83] employing multi-walled carbon nanotubes (MWCNTs)/water nanofluids, and as seen from this graph, the measured results are close to the fully developed friction factor *f* = 16/*Re* of the theoretical solution of circular tubes.

It can be seen that the current results and most of the experimental data agree with the analytical solution from Duan and Muzychka [76] within ±30%. To account for this discrepancy, a careful analysis of the experimental uncertainty is performed in this study. A basic equation of uncertainty analysis was presented by Moffat [85] utilizing the root sum square method and the equation can be expressed as

(23) $$U_{R}=\sqrt{{\sum_{i=1}^{n}\left(\frac{\partial R}{\partial x_{i}}U_{i}\right)}^{2}}$$

where *R* is the evaluation object, *x_i_* is a variable in *R*, and *U_i_* is the uncertainty of the variable *x_i_*. The friction factor can be expressed as follows

(24) $$f=\frac{\Delta PD_{h}}{2\rho u_{m}^{2}L}$$

According to Equation (10), the experimental uncertainties in *f* are given as

(25) $$\frac{U_{f}}{f}={\left[{\left(\frac{U_{\Delta P}}{\Delta P}\right)}^{2}+{\left(\frac{U_{D_{h}}}{D_{h}}\right)}^{2}+{\left(\frac{U_{\rho }}{\rho }\right)}^{2}+2{\left(\frac{U_{u_{m}}}{u_{m}}\right)}^{2}+{\left(\frac{U_{L}}{L}\right)}^{2}\right]}^{1/2}$$

From Equation (25), it is seen that the uncertainties in *f* are the measurements of the pressure drop, microchannel hydrodynamic diameter, density, flow velocity, and channel length. It is extremely difficult to accurately measure the dimension of microchannels, particularly when the dimensions reach microscale levels. In addition, entrance effects and channel surface roughness effects caused by the fabrication technique play an important role in the experimental uncertainties.

## 5. Conclusions

A three-dimensional numerical study of the flow characteristics of nanofluids in microchannel plate fin heat sinks considering entrance effects has been carried out in this paper. The effects of the Reynolds number, channel aspect ratio, and nanofluid volume fraction on pressure drop and entropy generation in microchannel plate fin heat sinks were analyzed in detail. In light of the preceding discussions, we draw the following conclusions.

(1) For given nanoparticle volume fraction and channel aspect ratio, the frictional entropy generation and pressure drop of the microchannel plate fin heat sinks dramatically increase by increasing the Reynolds number. For the 1 vol.% Al_2_O_3_–water nanofluid, when the Reynolds number increases from 500 to 1000, the pressure drop and frictional entropy generation of rectangular microchannel heat sinks with aspect ratio *ε* = 1 increases by 144% and 389%, respectively. In addition, the *f*_app_*Re* at low Reynolds numbers is higher than the values at high Reynolds numbers, especially in the entrance region.
(2) The frictional entropy generation and pressure drop slightly increases as the channel aspect ratio decreases. For the 1 vol.% Al_2_O_3_–water nanofluid, when the aspect ratio increases from 0.6 to 1 at *Re* = 500, the pressure drop of the channel decreases by 2%; however, when the aspect ratio increases from 0.1 to 0.5, the pressure drop of the channel decreases by 15%. The results also demonstrate that the flow parameters are not observably affected by the geometry of the cross-section at the entrance region.
(3) Given an increase in nanoparticle volume fraction, the frictional entropy generation and pressure drop have a slight increase. When the nanoparticle volume fraction increases from 0 to 1% at *Re* = 500, the pressure drop of microchannel plate fin heat sinks with the aspect ratio *ε* = 0.5 increases by 3%. Further, when the nanoparticle volume fraction increases from 1 to 5%, the pressure drop of the channel increases by 12%. The results mean that a heat transfer enhancement can be obtained by adding appropriate volume fraction of nanoparticles in a base fluid without significantly increasing pump work.
(4) The higher viscous entropy generation rate gradient and significantly higher *f*_app_*Re* values occur in the entrance region, which indicates the critical importance of the effect of the entry region in determining the behavior of microchannel heat sinks. Furthermore, the general expression of the entropy generation rate considering entrance effects is developed.

In future research work, we will further study the cooling performance of microchannel plate fin heat sinks with different nanofluids as coolants employing entropy generation minimization method.

## Figures and Tables

**Table 1:** Dimensions of the computational domain in present work.

ε	*a* (μm)	*b* (μm)	*D_h_* (μm)	*L* (mm)	Mesh (Computational Domain)
0.1	1100	110	400	14	700 × 165 × 17
0.2	600	120	400	14	700 × 90 × 18
0.3	433	130	400	14	700 × 65 × 20
0.4	350	140	400	14	700 × 53 × 21
0.5	300	150	400	14	700 × 45 × 23
0.6	267	160	400	14	700 × 40 × 24
0.7	243	170	400	14	700 × 37 × 25
0.8	225	180	400	14	700 × 34 × 27
0.9	211	190	400	14	700 × 32 × 28
1	200	200	400	14	700 × 30 × 30

**Table 2:** Thermophysical properties of base fluid (deionized water) and Al_2_O_3_ particles.

Material [Reference]	*ρ* (kg/m^3^)	*Cp* (J/kgK)	*k* (W/mK)	*μ* (Pa·s)	*d_p_* (nm)
Deionized water [39]	996	4178	0.611	0.000859	-
Al_2_O_3_ [70]	3380	765	30	-	47

**Table 3:** Simulation parameters of thermophysical properties of Al_2_O_3_–water nanofluid.

Nanofluids	*Φ* (%)	*ρ* (kg/m^3^)	*Cp* (J/kgK)	*μ* (Pa·s)
Al_2_O_3_-water	0.5	1007.92	4120.77	0.000870
Al_2_O_3_-water	1	1019.84	4064.88	0.000881
Al_2_O_3_-water	3	1067.52	3853.81	0.000927
Al_2_O_3_-water	5	1115.20	3660.79	0.000977

## Nomenclature

*a*	major semi-axis of rectangle, μm
*A*	flow area, m^2^
*b*	minor semi-axis of rectangle, μm
*Cp*	specific heat capacity at constant pressure, J/(kg·K)
*d_p_*	particle diameter, nm
*D_h_*	hydraulic diameter, = 4*A*/*P*
*f*	Fanning friction factor, = $\bar{\tau }/\left(\frac{1}{2}\rho u_{m}^{2}\right)$
*H*	height of heat sinks, mm
*k*	thermal conductivity, W/(m·K)
*L*	length of heat sinks, mm
*p*	pressure, N/m^2^
*Po*	Poiseuille number, = $\bar{\tau }D_{h}/\mu u_{m}$
*Pr*	Prandtl number,= μCp/k
$\dot{Q}$	heat transfer rate, W
*R*	evaluation object
*Re*	Reynolds number, = $u_{m}D_{h}/\nu$
${\dot{S}}_{\mathrm{gen}}^{\prime \prime \prime }$	local entropy generation rate, W/(m^3^·K)
${\dot{S}}_{\mathrm{gen}}$	global entropy generation rate, W/K
*t*	fin thickness, m
*T*	temperature, K
*u*	velocity, m/s
*u_m_*	average velocity, m/s
*U*	velocity scale, m/s
*U_i_*	uncertainty of the variable *x_i_*
*W*	width of heat sinks, mm
*x*, *y*	Cartesian coordinates, m
*x_i_*	independent variable
*z*	coordinate in flow direction, m
Greek symbols	
*ε*	aspect ratio, = *b*/*a*
*μ*	dynamic viscosity, N·s/m^2^
*ν*	kinematic viscosity, m^2^/s
*ξ*	dimensionless hydrodynamic channel length, = $L/\left(D_{h}Re\right)$
*ρ*	density, kg/m^3^
$\bar{\tau }$	wall shear stress, N/m^2^
ϕ	nanoparticle volume fraction, %
Subscripts	
*a*	ambient
*b*	heat sink base surface
*bf*	base fluid
*D_h_*	based upon the hydraulic diameter
*m*	mean
*nf*	nanofluid
*p*	nanoparticle
